# Indirect costs and public finance consequences of heart failure in Poland, 2012–2015

**DOI:** 10.1186/s12889-018-6034-0

**Published:** 2018-09-19

**Authors:** Błażej Łyszczarz

**Affiliations:** Department of Public Health, Faculty of Health Sciences, Nicolaus Copernicus University in Toruń, ul. Sandomierska 16, 85-830 Bydgoszcz, Poland

**Keywords:** Heart failure, Indirect costs, Human capital method, Poland, Cost-of-illness, Productivity losses

## Abstract

**Background:**

As a consequence of unfavourable epidemiological trends and the development of disease management, the economic aspects of heart failure (HF) have become more and more important. The costs of treatment (direct costs) appear to be the most frequently addressed topic in the economic research on HF; however, less is known about productivity losses (indirect costs) and the public finance burden attributable to the disease. Therefore, the aim of this study was to estimate the indirect costs and public finance consequences of HF in Poland in the period 2012–2015.

**Methods:**

The study uses a societal perspective and a prevalence-based top-down approach to estimate the following components of HF indirect costs: absenteeism of the sick and their caregivers, presenteeism of the sick, disability, and premature mortality. The human capital method has been chosen to identify the value of productivity losses attributable to HF and the public finance consequences of the disease. Deterministic sensitivity analysis was performed to assess the robustness of the results.

**Results:**

The total indirect costs of HF in Poland were €871.9 million in 2012, and they increased to €945.3 million in 2015. In the period investigated, these costs accounted for 0.212–0.224% of GDP, an equivalent of 22.63€–24.59€ per capita. Mortality proved to be the main driver of productivity losses, with 59.3–63.4% of the total costs depending on year, followed by presenteeism (21.1–22.5%), disability (11.1–14.2%) and the sick’s absenteeism (3.3–4.0%). The cost of caregivers’ absenteeism was unimportant. The social insurance expenditure for benefits associated with HF accounted for €40.7 million in 2012 and €45.6 million in 2015 (0.56–0.59% expenditure for all diseases). The potential public revenue losses associated with HF were €262.7–€287.9 million. Sensitivity analysis showed that the costs varied by − 12.1% to + 28.8% depending on the model parameter values.

**Conclusion:**

HF is a substantial burden on the economy and public finance in Poland. By confronting the disease more effectively, the length and quality of life for those affected by HF could be improved, but society as a whole could also benefit from the increased economic output.

## Background

Heart failure (HF) is a cardiovascular syndrome that affects millions worldwide and is the subject of growing attention on the part of the health care community. HF is a rising concern particularly in developed countries where the disease is increasingly widespread as a result of population ageing and improvements in the survival of patients with cardiovascular conditions [1, 2]. Epidemiological trends and advancement in cardiovascular disease management predict dramatic growth in the health and economic burden of HF [3]. In Poland, approximately 0.7 million people are directly affected by HF [4], and the disease is the 3rd and 6th most important cause of years of life lost among women and men, respectively [5]. Compared to the European population, Polish patients develop the disease at a younger age; they are also more often hospitalized [6] and less capable of self-care [7].

Due to the unfavourable epidemiologic trends and the development of disease management, the economic aspects of HF have become more and more important. The costs of treatment (direct costs) appear to be the most widely addressed topic in the economic research on HF, as from the examples of Germany [8], the United Kingdom [9], the United States [10] and Poland illustrate [11]. Estimates from Poland show that the public payer’s expenditure for HF treatment in 2011 exceeded €410 million, 3.2% of the payer’s budget [11], while the annual cost of HF identified in a recent systematic review comprising the whole world ranged from 908 to 40,971 international dollars per patient [12]. The costs of treatment are important for both payers and patients; however, the negative economic consequences of HF spread beyond the direct costs. The disease leads to productivity losses (indirect costs) attributable to employee work absence, disability and premature mortality. Additionally, the efficiency of the sick and their caregivers declines even if they continue to work. Furthermore, higher expenditure is demanded of public funds to secure social benefits for those affected by the disease. These aspects of HF economics are less frequently explored, but as the scarce evidence suggest, indirect costs contribute significantly to the overall burden caused by the disease. According to Spanish estimates, informal caregiving costs constitute 59.1% to 69.8% of total HF costs [13]; an American study reports HF mortality costs of $10.6 billion [14], while a study from Nigeria approximates the work absence costs of 239 HF cases from a tertiary hospital at an equivalent of US$254238, 44.3% of the overall costs [15]. These studies give important insights into the knowledge on productivity losses attributable to HF; however, the burden estimated therein only accounts for selected components of indirect costs such as mortality, caregiving or work absence in separation, and they do not provide a broad view on total losses. Another American study shows a more comprehensive picture of HF indirect costs, reporting on morbidity losses among those employed and disabled as well as losses in home production and those due to mortality [16]. This study’s findings seem to be the only ones comparable to the present study in terms of the cost components included; however, the American estimates are based on medical expenditure survey data, while here I mostly rely on social insurance statistics. Moreover, until now, no study has reported on the public finance burden of HF.

Therefore, the purpose of this study is to fill the gap in knowledge on the HF economic burden by estimating the productivity losses and public finance consequences resulting from the disease in Poland. This research’s contribution is as follows. It is the first study that attempts to estimate a wide range of indirect costs for a European country, including not only the sick’s absenteeism, disability and mortality costs but also losses caused by their presenteeism and informal carers’ absenteeism. Second, the estimates provide insight into the public finance significance of HF, both in terms of expenditure on social benefits and potential public revenue decline. Finally, based on a four-year period, the study gives a dynamic picture of the economic burden of HF.

## Methods

The study uses a societal perspective and prevalence-based top-down approach to estimate the indirect costs of HF in Poland in the years 2012–2015. The human capital method (HCM) is applied to assess the costs of absenteeism of the sick and their caregivers; presenteeism of the sick; and premature mortality and disability caused by HF (ICD-10 code: I50). For each of these categories, I estimated the total time not worked or worked with decreased productivity and the value of production lost because of the disease.

Gross domestic product (GDP) per worker was used as a measure of productivity. For farmers’ population, I adopted this measure adjusted for lower productivity in the agricultural sector (per worker gross value added in the agricultural sector was as low as 17.2% of per worker gross value added in the Polish economy, and this value was used to correct for productivity in the agricultural sector). The estimates accounted for decreasing marginal labour productivity. This was done by adjusting the productivity losses of each cost component with a 0.65 correction coefficient, which approximates the output elasticity of labour in the production function [17, 18]. A sex-specific retirement age (60 for females and 65 for males) was used.

Table 1 presents details on the main parameters used in estimating the costs of HF, and the following paragraphs explain the basic assumptions of calculating losses associated with particular cost categories. A more detailed description of the methodological approach used here is available in a study concerned with the costs of breast cancer [18].

**Table 1:** Main parameters of the model for estimating indirect costs of heart failure in Poland, 2012–2015

	Parameter (unit)	Mean value for years 2012–2015
General economic parameters		
Gross domestic product (€)		406,258,566 346^a^
Per worker gross domestic product (€)		28 745^a^
Correction coefficient to adjust for decreasing marginal labour productivity		0.65
Exchange rate (zlotys per €)		4.19
Male and female retirement age (years)		65/60
Economy’s yearly productivity growth for period 2016-2077^b^		1.9%
Parameters for estimating indirect costs		
Absenteeism of the sick	Number of absence days	476 598^c^
	Number of people receiving first-time rehabilitation benefits	213
	Average duration of first-time rehabilitation benefits (months)	5.95
	Number of people receiving renewed rehabilitation benefits	111
	Average duration of renewed rehabilitation benefits (months)	5.33
Presenteeism of the sick	Number of sick people at working age	214 047^d^
	Employment rate of people at working age affected by HF [20]	23%
	Rate of productivity reduction while working [29]	22.7%
Caregivers’ absenteeism	Number of absence days due to relative’s illness	2473
Premature mortality	Number of HF deaths from birth to retirement age	5504
Disability	Number of people receiving disability pensions^e^	
	● permanent pension	640
	● temporary pension	4208
	Average duration of temporary disability pension in cardiovascular diseases (months)	16.9
	1-year HF survival rate [21]	89.2%^f^

Notes: ^a^ – values in Euro (€) calculated using constant average 2012–2015 exchange rate: 4.19 zlotys per €; ^b^ – the timespan covers the period of potential economic activity of the youngest person who developed HF during the period investigated; based on [30]; ^c^ – for population insured in ASIF the data for 2012 and 2013 were interpolated using data from the subsequent 3 years; ^d^ – the value estimated based on the age distribution of the American population with HF [19]; ^e^ – the values show an equivalent of people who are completely unable to work assuming that partial inability to work corresponds to 0.75 of complete inability to work. For the population insured in SII, real numbers are used; for those insured in ASIF, a part of the figures were estimated; ^f^ – the value refers to both sexes and only the hospitalized population

In this study, the sick’s absenteeism refers to short- and medium-term absence from work. It was approximated by a number of absence days recorded by two social insurance institutions that insure the general population (SII – Social Insurance Institution) and farmers (ASIF - Agricultural Social Insurance Fund); and by a number and duration of rehabilitation benefits issued in the case of an absence longer than 180 days but with an expected return to work afterwards. Estimating the magnitude of presenteeism required identifying the number of those affected by HF being of working age and professionally active. Because age-specific prevalence of HF in Poland is not available, I used US data [19] to approximate the number of HF population of working age. According to Polish estimates, the employment rate of people of working age affected by HF was 23% [20]. By subtracting disability pensions and rehabilitation benefits, I obtained the number of those working despite their condition. Further, the number of absence days was subtracted, resulting in the time worked by the HF population. Using the only estimate of productivity reduction in HF found, I applied a 22.7% rate as a measure of presenteeism magnitude in this disease [21]. Caregivers’ absenteeism was assessed with social insurance data based on the estimated absence due to informal care provided to an adult relative. In the absence of disease-specific data for this cost component, I assumed that the share of absence certificates issued for HF caregivers is the same as the HF share of own sickness absence, for which data are available.

For premature mortality costs, I used data on the number of HF sex-specific deaths in 5-year age groups [22] and assumed that the distribution of deaths within these groups was the same as in the total mortality in Poland. In this way, the number of deaths at each working age was identified, and after adjusting for probability of future death from other causes and employment rate, I approximated the number of premature deaths (at each working age) of those affected by HF who would have worked if not deceased. The product of this number and the age-specific discounted value of potential production lost for an HF person at every age until retirement resulted in the total indirect cost of premature mortality being obtained. The costs of disability were assessed with data from two social insurance institutions, SII and ASIF. Inability to work in Poland is classified in two dimensions, according to its duration and degree. Using this classification and data obtained from insurers, I identified the number of people with HF receiving permanent/temporary and complete/partial disability pensions. For each of the four categories, I estimated the number of people receiving particular benefits, average time of pension duration and a discounted value of production lost at every age. The sum of the losses for each pension category approximated the costs of disability related to HF.

The public finance consequences of HF were identified in two areas. First, I summed up the expenditure of SII for social benefits related to the disease. Second, I estimated public revenue losses due to reduced economic output attributable to HF. To estimate these losses, I calculated the shares of four main taxes (VAT, personal and corporate income taxes; and the excise tax, which together contribute 89% of overall central budget revenues) and social insurance contributions in GDP and multiplied these shares by the indirect costs of HF; the sum of these products represents potential public revenue decline attributable to the disease. The public finance analysis did not account for direct costs of HF because data on cost of treatment was not available for the period investigated.

One-way deterministic sensitivity analysis was carried out to test the stability of the results. Variation in model parameters was restricted to year 2015 solely and included:

- using 0% and 3.5% discount rates;
- using minimum and maximum exchange rates from the period investigated instead of the mean rate;
- ±0.05 variation in the coefficient, which accounts for decreasing labour productivity;
- ±20% change in the rate of productivity reduction in presenteeism;
- ±40% variation in the number of caregivers’ absence days;
- ±20% in the number of people with HF at working age;
- using gross value added instead of GDP as a productivity measure;
- decreasing number of deaths due to HF by 20%.

Because this study does not involve any participants and relies only on data collected for other purposes, the ethics committee approval was not required.

## Results

### Indirect costs estimates

The total indirect cost (productivity losses) of HF in Poland was €871.9 million in 2012, and this increased to €945.3 million in 2015. In the period investigated, the dynamics of HF indirect costs (8.4–percent raise) was lower than the dynamics of GDP (10.4–percent increase). The highest costs were those of premature mortality, with the burden ranging from €517.4 million to €581.7 million depending on the year. Presenteeism of the sick generated substantial losses (€193.6 – €199.3 million), followed by disability (€96.5 to €126.3 million) and absenteeism of the sick (€28.8 – €37.9 million). The magnitude of caregivers’ absenteeism was negligible, with costs of €0.2 million at most. The production lost due to HF in Poland accounted for approximately 0.22% of GDP throughout the period; this share declined slightly in the years 2013–2014, followed by an increase in 2015. The indirect costs corresponded to 22.63€–24.59€ per capita (Table 2).

**Table 2:** Indirect costs of heart failure in Poland, 2012–2015

		Absenteeism of the sick	Presenteeism of the sick	Caregivers’ absenteeism	Premature mortality	Disability	Total
2012	Total cost (€)	28,772,687	193,613,105	157,145	552,882,342	96,466,310	871,891,590
	% of GDP	0.0074	0.0498	< 0.0000	0.1421	0.0248	0.2241
	Costs per capita (€)	0.75	5.02	0.00	14.35	2.50	22.63
2013	Total cost (€)	31,773,141	194,451,069	171,172	524,283,629	122,034,015	872,713,026
	% of GDP	0.0080	0.0491	< 0.0000	0.1325	0.0308	0.2205
	Costs per capita (€)	0.83	5.05	0.00	13.62	3.17	22.67
2014	Total cost (€)	34,752,086	195,710,394	182,379	517,424,137	123,864,968	871,933,964
	% of GDP	0.0085	0.0477	< 0.0000	0.1260	0.0302	0.2123
	Costs per capita (€)	0.90	5.09	0.00	13.45	3.22	22.66
2015	Total cost (€)	37,891,329	199,251,868	227,537	581,708,479	126,255,787	945,335,000
	% of GDP	0.0088	0.0464	0.0001	0.1354	0.0294	0.2200
	Costs per capita (€)	0.99	5.18	0.01	15.13	3.28	24.59

Source: own estimates. Notes: Total cost values in Euro currency calculated using constant average 2012–2015 exchange rate: 4.19 zlotys per €

**Fig. 1:**
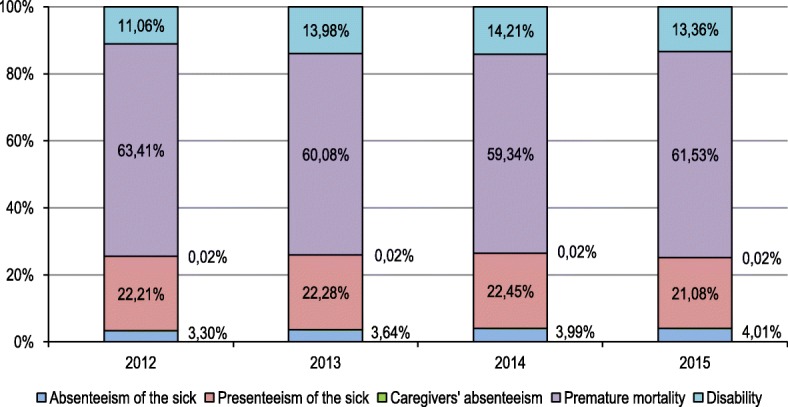
Structure of indirect costs categories in heart failure in Poland, 2012–2015. Notes: the value of 0.02% on the right from each bar refers to caregivers’ absenteeism which is too low to be readable directly from the figure

Among the five cost categories, premature mortality was the main driver of productivity losses, accounting for 59.3% to 63.4% of the total costs. Presenteeism amounted for 21.1% to 22.5% of the total burden; losses due to disability ranged from 11.1 to 14.2% of the costs, and the sick’s absenteeism caused 3.3–4.0% of the productivity reduction due to HF. The share of caregivers’ absenteeism in the total costs was 0.02% in each year. The importance of the sick’s absenteeism in total costs grew year over year. On the other hand, the dynamics of other indirect cost components was instable over time (Fig. 1).

### Public finance consequences

The expenditure of SII (the institution that provided social insurance to 91.5% of the insured population in 2015) for benefits associated with HF accounted for €40.7 million in 2012 and €45.6 million in 2015, a change of 12.2%. These values corresponded to 0.56–0.59% of expenditure for all diseases. A majority of these expenses was disability pensions, which constituted more than 85% of the total HF benefits expenditure over the period. The other category, with a more than 10% share in total spending, was sickness benefits, and these payments increased by 35.1% from 2012 to 2015. The dynamics of other categories were even higher, although their magnitude in benefits structure was low, with less than €1 million spending per year for each one (Table 3).

**Table 3:** Social insurance expenditures for benefits associated with heart failure in Poland, 2012–2015 (€)

	2012	2013	2014	2015
Rehabilitation benefits	564,914	708,642	888,345	934,120
Medical rehabilitation within the framework of disability prevention	38,134	40,874	68,029	78,607
Disability pensions	35,420,237	38,363,852	39,050,670	37,976,289
Social pension	252,116	296,775	376,251	692,065
Sickness benefits	4,376,913	5,023,673	5,169,202	5,912,987
Total expenditures for HF benefits (% of expenditure for all diseases)	40,652,315 (0.56)	44,433,816 (0.58)	45,552,496 (0.59)	45,594,069 (0.56)

Source: data obtained from SII. Notes: Data refers only to SII’s expenditure; data for ASIF on benefits for farmers population were not obtainable. All values in Euro currency calculated using constant average 2012–2015 exchange rate: 4.19 zlotys per €

The estimate of the potential public revenue losses associated with HF shows that (state and regional) budgets, and social insurance funds lost €262.7 million in 2012 and €287.9 million in 2015 because of the disease. The losses in social insurance contributions dominated the public finance burden, with €112.0 – €127.7 million of revenues lost; among taxes, decreased inflows in VAT and PIT were the highest (Table 4).

**Table 4:** Potential losses in state and regional budgets and social insurance fund revenues due to productivity reduction attributable to heart failure in Poland, 2012–2015

	B: Public finance revenue losses due to heart failure^a^ (€)			
	2012	2013	2014	2015
VAT	63,710,089	62,355,192	60,781,641	65,790,475
Excise tax	32,172,789	31,856,042	31,190,101	33,427,687
Corporate income tax (CIT)	16,738,061	15,885,989	15,485,829	16,937,035
Personal income tax (PIT)	38,054,395	38,772,429	39,569,917	44,021,527
Social insurance contributions. Incl.	111,987,846	115,154,912	116,486,628	127,709,256
- health insurance contributions	32,743,572	32,853,179	32,609,080	35,320,322
Total	262,663,179	264,024,563	263,514,116	287,885,979

Notes: ^a^ – values in Euro currency calculated using constant average 2012–2015 exchange rate: 4.19 zlotys per €

### Sensitivity analysis

The results of sensitivity analysis show that the variation of the HF indirect costs in Poland differ depending on the parameters applied. With no discounting, the total costs proved to be 28.8% higher than in the base scenario, while using a 3.5% discount rate increased the costs by 6.5%. Variation in the exchange rate changed the results by − 7.2% to 5.2%, and a change in the value of the labour productivity correction coefficient by ±0.05 led to a ± 7.7% change in the overall loss. By a ± 20% change in the number of people with HF, I obtained a ± 4.5% change in the costs, and the effect of a one-fifth change in the rate of productivity reduction in presenteeism resulted in ±4.2% variation. Productivity losses proved to be lowest when using gross value added as a productivity measure (− 11.4%) and when decreasing the number of deaths by 20% (− 12.1%) (Table 5).

**Table 5:** Sensitivity analysis for estimates of heart failure indirect costs in Poland (2015) according to varying assumptions regarding model parameters

	Total indirect costs (€)	Change from base scenario
Base scenario (BS)	945,335,000	–
Discount rate (BS: 5%)		
0%	1,217,148,974	28.8%
3.5%	1,007,078,204	6.5%
Exchange rate (BS: 4.19 zlotys per €)		
3.98	994,166,151	5.2%
4.51	877,139,348	− 7.2%
Coefficient to adjust for decreasing marginal labour productivity (BS: 0.65)		
0.6	872,616,923	− 7.7%
0.7	1,018,053,077	7.7%
Rate of productivity reduction for presenteeism of the sick (BS: 22.7%)		
18.2% (− 20%)	905,484,627	− 4.2%
27.2% (+ 20%)	985,185,374	4.2%
Number of caregivers’ absence days (BS: 2966)		
1780 (−40%)	945,244,014	0.0%
4152 (+ 40%)	945,425,979	0.0%
Productivity measure (BS: Per worker gross domestic product)		
Per worker gross value added	837,361,164	− 11.4%
Number of people with HF at working age (BS: 212987)		
170,389 (−20%)	902,511,375	−4.5%
255,584 (+ 20%)	988,158,626	4.5%
Number of deaths due to HF (BS: 5633)		
4506 (−20%)	830,900,512	−12,1%

## Discussion

Based on a social perspective and prevalence-based top-down approach, this study approximates the economic burden of HF in Poland in the period 2012–2015. This is the first European study that estimates a wide range of productivity loss components, including absenteeism of the sick and their caregivers; presenteeism of those affected by HF; premature mortality; and disability. The other contributions of this paper include an assessment of the consequences of HF for public finance in terms of social expenditures and decreased public revenues; in addition, the dynamics of the disease’s economic consequences are explored.

The results show that HF in Poland accounted for indirect costs of €871.9 million in 2012 and grew to €945.3 million in 2015, generating an 8.4% increase. The productivity losses due to HF were 25% higher than those associated with breast cancer in Poland [18] but lower than in the case of diabetes (2 billion US$) [23] (2014 estimates for all three diseases). The production lost due to HF was an equivalent of 0.212–0.224% of GDP, and no clear time trend was observed; the costs declined in 2012 and 2013 and then increased in 2014. Most of the costs were attributable to premature deaths of HF patients, followed by presenteeism of the sick and disability. The magnitude of mortality losses in HF was notable and much higher than in breast cancer (61.1% vs. 23.5% on average) [18]; this result needs to be interpreted with caution, however, because heart failure is considered a “garbage code” in registering death causes [24]. Moreover, reporting cause-specific mortality in Poland is of questionable quality, and the number of deaths reported due to HF is most likely inflated; however, a vast majority (87%) of the deaths classified with “garbage code” in cardiovascular diseases refer to the population aged 65+ [25], which is not a subject of interest here because it is professionally inactive. The magnitude of the sick’s absenteeism was relatively low; however, it was increasing steadily during the period analysed. The production lost due to caregivers’ absence was negligible, but this result appears to be understated. The calculation of this cost component used the share of HF own absence in all-cause own absence to approximate caregivers’ absence, and because HF largely affects those who are retired, own absence is comparatively low in this case. Moreover, using data on short-term absence, I was not able to identify the losses associated with leaving the labour market by those caregivers who decided to provide care permanently. The difficulties with estimating indirect costs of caregiving show that this cost category requires focus on conceptualization and data collection improvements to achieve more reliable results.

The results show the substantial public finance burden of HF in Poland. The social benefits expenditure related to HF increased by 12.2% (from €40.7 to €45.6 million) during the 4-year period, which is more than the rise of indirect costs (8.4%) and social spending for all diseases (11.2%). Expenditure for each benefit category increased year over year, including disability pensions, and the dynamics of this category were the opposite to that of breast cancer, wherein public finance expenditure decreased over time [18]. Decreased taxes and social insurance revenues accounted for €262.7 to €287.9 million, yielding a 9.6% increase. Altogether, the high dynamics of the public revenue losses attributable to HF show that the public sector in Poland was more susceptible to the economic burden of the disease than the general economy. Because of data constraints, the public finance consequences of HF analysed in this paper did not include three important categories that should be a part of a complete public cost analysis. These include the following: the health care cost of HF treatment; the foregone spending on retirement pensions; and the foregone VAT of the retired. The costs of HF treatment in Poland estimated elsewhere show that this category alone accounted for more than €400 million in 2011, which was 3.16% of the public payer’s budget [11]. Therefore, it must be kept in mind that the public finance burden identified here only accounts for expenditure on social benefits and foregone tax revenues of public budgets and does not include the cost of health care.

Sensitivity analysis showed that my estimates are fairly susceptible to the changes in model parameters. Varying model parameters led to estimates differing from the base scenario by − 12.1% to + 28.8%, and this variation is mainly attributable to a high share of premature mortality in the total costs. Because this cost category accounts for > 60% percent of the overall burden and, at the same time, death’s consequences extend into the future, with no discounting, we observe the relatively highest variation of the total costs (+ 28.8%). Similarly, when decreasing the number of deaths by 20%, to account for over-registration of HF deaths, the indirect costs estimate responds notably (− 12.1%).

The estimates from this study are comparable with other findings to only a limited extent. In fact, it appears that only the American research [14, 16] reports HF consequences for the whole economy. The studies using data from Spain [13] or Nigeria [15] estimate the costs for patient samples and do not generalize the results to the country level. The study from the US reports the indirect costs of HF mortality in 2010 as $10.6 billion [14], an equivalent of 0.071% of GDP, which is approximately half the GDP share of the present estimate for mortality costs in Poland. This discrepancy may arise from a variety of differences across the study methods and country settings, including the productivity measures and discount rates used; retirement age; or age distribution of deaths. Interestingly, the other study from the US reported similar indirect costs of $9.8 billion in 2012 [16], as in the first American study mentioned [14]; however, this last estimate accounted not only for mortality costs but also for absenteeism, disability and home productivity. The scarcity of results on HF indirect costs combined with the ambiguous findings from previous studies call for action in developing a framework that would enhance comparability of future results. Thus, to validate the productivity losses attributable to HF estimated so far, studies from other countries are necessary.

This study has certain limitations. First, although it comprises several cost categories not included in previous studies, it still lacks estimates for caregivers’ presenteeism, housekeeping activities and intangible costs. Because of data unavailability, I was not able to include these components in the productivity loss assessment, and for this reason, my results are underestimated. On the other hand, HCM, which is used here to identify costs, is sometimes thought to overestimate losses because it results in estimating the maximum burden of diseases. For this reason, HCM is subject to criticism [26, 27]; however, there is no agreed alternative, and HCM remains the most common approach in applied research. The other limitation is that in some cases, it was necessary to rely on estimated data (e.g., number of deaths at a particular age; disability in farmers’ population) or figures from other countries (e.g., age-specific prevalence; rate of productivity reduction in presenteeism). This might bias the estimates, and thus, caution is necessary in interpreting the findings. This drawback is not unique for the present study, however, and numerous other papers use a similar approach in estimating indirect costs of diseases. A similar limitation arises from using an average productivity measure (GDP per worker), which does not account for the potential deviation from mean productivity in the economy of those affected by HF. This fact may overestimate the costs because HF is associated with low socioeconomic status [28], implying that the productivity of the sick may be lower than average. Unfortunately, this effect could not be corrected for with no available data on the socioeconomic status of the HF working population. Finally, with the low quality of cause-specific death registers in Poland, one must be careful with the estimates of mortality costs. To address this shortcoming, I tested the validity of the findings in sensitivity analysis.

## Conclusions

In conclusion, HF is a substantial burden both for the whole economy and public finance in Poland. Although the disease largely affects older citizens who are no longer professionally active, the productivity losses associated with HF in the working population account for 0.22% of GDP, a high share compared to the US estimates discussed above [14, 16]. This large burden in Poland arises from the relatively high mortality of those who are of working age, as the cost of HF deaths accounts for approximately 60% of total costs. It is noteworthy that the productivity losses attributable to HF are approximately twice as high as the direct costs borne by the public payer for treatment of the condition [11]. This suggests that confronting the disease more effectively would not only improve the length and quality of life for those affected by HF but that society as a whole might benefit from the increased economic output and improved public finances.

## Abbreviations

ASIF: Agricultural Social Insurance Fund
BS: Base scenario
CIT: Corporate income tax
GDP: Gross domestic product
HCM: Human capital method
HF: Heart failure
PIT: Personal income tax
SII: Social Insurance Institution
VAT: Value added tax
